# A Deep Learning Approach on Building Detection from Unmanned Aerial Vehicle-Based Images in Riverbank Monitoring

**DOI:** 10.3390/s18113921

**Published:** 2018-11-14

**Authors:** Wuttichai Boonpook, Yumin Tan, Yinghua Ye, Peerapong Torteeka, Kritanai Torsri, Shengxian Dong

**Affiliations:** 1 School of Transportation Science and Engineering, Beihang University, Beijing 100191, China; wuttichai@buaa.edu.cn (W.B.); yhye@buaa.edu.cn (Y.Y.); 2 National Astronomical Observatories of Chinese Academy of Sciences (NAOC), University of Chinese Academy of Science, Beijing 100012, China; peerapong@nao.cas.cn; 3 Hydro and Agro Informatics Institute (HAII), Ministry of Science and Technology, Bangkok 10400, Thailand; kritanai@haii.or.th; 4 International Center for Climate and Environment Sciences (ICCES), Institute of Atmospheric Physics (IAP), University of Chinese Academy of Science, Beijing 100029, China; 5 Remote Sensing Center, Yangtze Normal University, Chongqing 408000, China; 201107081106@stu.yznu.cn

**Keywords:** building extraction, UAV dataset, deep learning, river bank monitoring

## Abstract

Buildings along riverbanks are likely to be affected by rising water levels, therefore the acquisition of accurate building information has great importance not only for riverbank environmental protection but also for dealing with emergency cases like flooding. UAV-based photographs are flexible and cloud-free compared to satellite images and can provide very high-resolution images up to centimeter level, while there exist great challenges in quickly and accurately detecting and extracting building from UAV images because there are usually too many details and distortions on UAV images. In this paper, a deep learning (DL)-based approach is proposed for more accurately extracting building information, in which the network architecture, SegNet, is used in the semantic segmentation after the network training on a completely labeled UAV image dataset covering multi-dimension urban settlement appearances along a riverbank area in Chongqing. The experiment results show that an excellent performance has been obtained in the detection of buildings from untrained locations with an average overall accuracy more than 90%. To verify the generality and advantage of the proposed method, the procedure is further evaluated by training and testing with another two open standard datasets which have a variety of building patterns and styles, and the final overall accuracies of building extraction are more than 93% and 95%, respectively.

## 1. Introduction

Heavy rainfalls can lead to widespread flooding along riverbank areas, especially in China nearly every summer, and Chongqing City suffers from flooding due to the Yangtze River that flows through it. Unmanned Aerial Vehicle (UAV)-based photogrammetry has been proven a competitive way to collect timely data in riverbank areas for its speed, convenience, and also its low cost in getting high-resolution images, high-accuracy orthoimages, and for 3D mapping [1,2], so it is very suitable for riverbank monitoring in Chongqing which is a mountainous city with a high population density and where is also cloudy most of the year.

However, for building detection and extraction from UAV images, building details will largely affect the detection accuracy in the case of very high spatial resolution. While various simple and complex building patterns could be easily interpreted by visual inspection, buildings in remotely sensed imagery may have various patterns and styles, which will cause a big problem. Traditional remote sensing image processing approaches, like classification methods (e.g., Maximum Likelihood Classifier (MLC) and Support Vector Machine (SVM)) [3], do not perform well on UAV images, because these methods require training samples to predict the synonymous features and they can only achieve good classification performance when certain criteria are met, such as a Gaussian distribution of data. The processing time is also a problem when the training samples are large. These limitations can be solved by using an Artificial Neural Network (ANN) [4]. Modern forms of ANN have been introduced, in particular Deep Learning (DL), which has already shown great performance in remote sensing applications such as scene classification, object detection, and semantic segmentation. In recent years, DL combined with UAV technology has been applied to many applications such as car detection [5,6], real-time scene understanding [7], location identification and core fire area segmentation [8], etc. DL can take advantage of spectral, textural, geometrical, and contextual features in classifying UAV images with much higher accuracy compared to object-based classification [9]. Zeggada et al. [10] proposed a Convolution Neural Network (CNN) model combined with multilabeling layer (ML) consisting of a customized thresholding operation to classify a grid of tiles based on multi-labelled UAV images. As to semantic segmentation, Kemker et al. [11] evaluated simple classification algorithms (k-nearest neighbor (kNN), linear support vector machine (SVM), multi-layer perceptron (MLP), spatial mean-pooling (ML)), spatial-spectral feature extraction methods (MICA and SCAE), and two Fully Connected Network (FCN) models (SharpMask and RefineNet) with multispectral UAV dataset (RIT-18), and their results showed that DL with RefineNet had the best performance compared to other classification techniques. Liu et al. [9] compared the performance of traditional object-based classifications (SVM and Random Forest (RF)) with Deep CNN (DCNN) on ortho-images and multi-view UAV images, and the results showed that the DCNN provided more classification accuracy than other traditional approaches, and the accuracy increased more obviously when multi-view data was used. Furthermore, Nogueira et al. [12] evaluated some typical DL semantic segmentation methods, including pixel-wise (standard ConvNet), fully convolutional (Fully ConvNet), deconvolutional (SegNet), and the combination of these methods called ensemble of ConvNets, and final conclusion was that ConvNets reached the highest overall accuracy of 96%. As a robust and accurate technique, DL semantic segmentation shows great performance on extracting objects from UAV images, and multi-view images can contribute significant improvement compared to orthoimage only.

Moreover, various network architectures have been proposed in DL semantic segmentation to improve building extraction accuracy, such as single patch-based CNN architecture [13,14], FCN [15], encoder-decoder network architecture [16,17]. Xu et al. [18] improved building extraction accuracy using a new model based on deep residual network (ResNet) which is defined as Res-U-Net and object-oriented guided filter, and the reported accuracy is around 5% higher than other standard models (SegNet, FCN). For better DL application in UAV image processing, Zhuo et al. [19] proposed a DL semantic segmentation on oblique images from UAV to optimize building footprint extraction from OpenStreetMap (OSM).

DL methods require huge training samples and the data complexity should be considered in the supervised learning process. Buildings along the Yangtze riverside in Chongqing have a variety of patterns and styles, while the buildings in the few public standard photogrammetry datasets currently published online have different styles, because they are collected from different geographic locations, so in our work, a set of building samples on the riverbank area is created and labeled for the supervised learning process and then DL semantic segmentation using the standard network architecture SegNet is evaluated. Moreover, the generality and advantage of proposed DL procedure are further verified by the application to two other standard datasets.

## 2. Materials and Methods

### 2.1. Study Area and Training UAV Datasets

**Figure 1:**
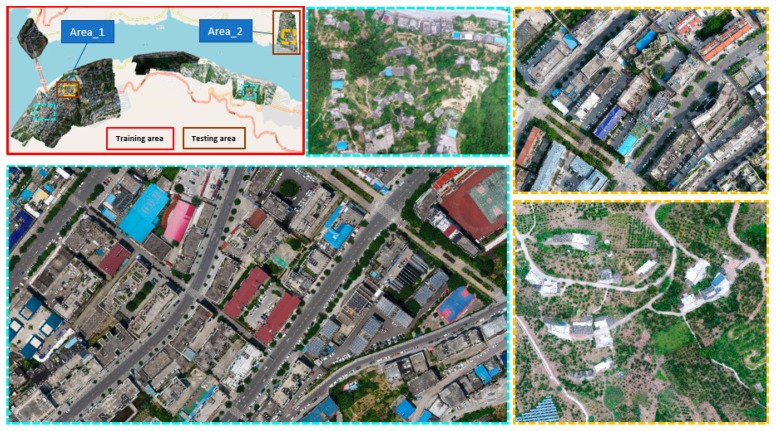
The study area; outer large red box in top left shows the entire study area; enlarged cyan boxes refers to training set area and yellow boxes are two testing areas.

The study area is located in the Fuling District of Chongqing City (China). Figure 1 shows the UAV data collection area overlayed on a Google Street map. The UAV used in this paper is a Quad-Rotor UAV (DJI Phantom 4 Pro, Shenzhen Dajiang Baiwang Technology Co., Ltd., Shenzhen, China) equipped with onboard capabilities, a positioning system (GPS/GLONASS), and DJI camera. The camera contains a CMOS sensor (13.2 mm × 8.8 mm) with 19.96 MP (megapixel) effective pixels: and a lens with a FOV (Field of View): 84°, focal length: 8.8 mm/24 mm (35 mm format equivalent), aperture: f/2.8–f/11, and focus distance (auto focus): 1 m to ∞. Raw images are stored as RGB mode (3:2 Aspect Ratio: 5472 × 3648 pixels) in JPEG format. All the UAV images used here were collected from 18 flights covering both the urban and countryside area. Lighting conditions, capture view, and flying parameters are not always the same for images not collected during the same flight, so these images represent a fairly good generalization. The entire object area is divided into training areas (red boxes), which cover different types of buildings, and testing areas (brown boxes) which are at different sites from the training areas. The testing areas are further separated into two areas according to different urban structures (Area_1 and Area_2).

**Figure 2:**
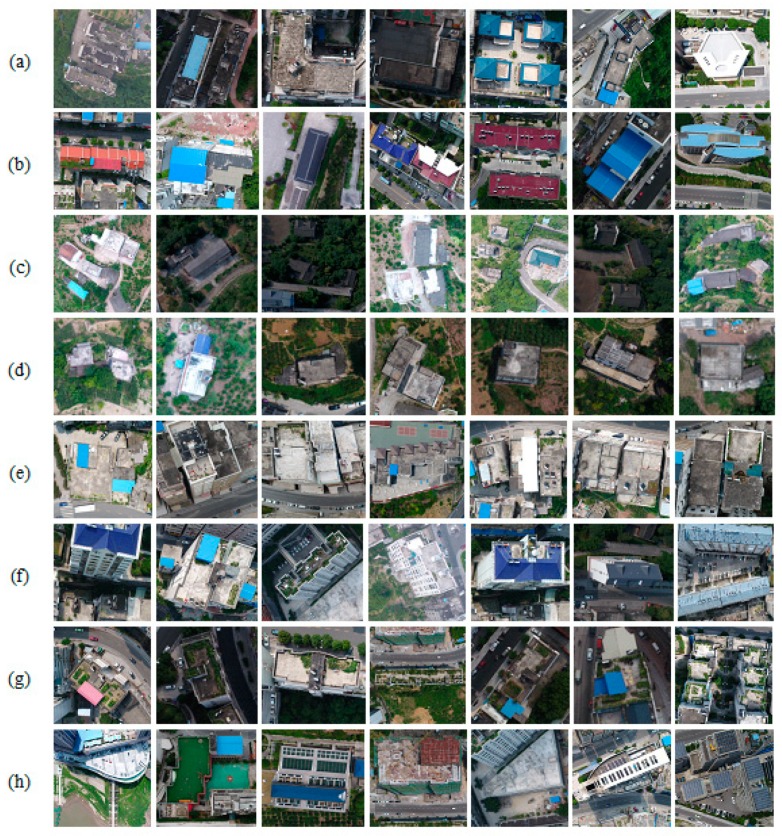
Buildings with different perspectives in study area. (**a**) building architectures; (**b**) high and medium-size building rooftops; (**c**) small-size building rooftops; (**d**) small building rooftops; (**e**) dense and tall buildings; (**f**) different sides of a building; (**g**) greening on building roofs; (**h**) playground on building roofs.

From Figure 1, we can see that there are various building patterns, architectures, scene characteristics, illumination condition, and styles in the entire study area. Figure 2 shows the buildings in object area are very complicated, and they may be grouped according to different perspectives, such as building architectures: (a), high and medium size building rooftops (b), small size building rooftops (c), small building rooftops (d), dense and tall buildings (e), different side of a building (f), greening on building roofs (g), and playground on building roofs (h). These simple or complex building patterns can be easily interpreted by visual inspection, while it is not easy for a machine learning method. Although there are already some open standard datasets provided online, such as AID, NWPU-RESISC45, PatternNet, RSI-CB256, RSSCN7, UCMerced_Landuse, WHU-RS19, Inria Aerial Image Labeling, ISPRS semantic labeling datasets, etc. which could represent many building types around the world, they are mostly captured from very high spatial resolution imagery on aerial or satellite platforms, and the training distribution of these open datasets is different from the distribution of this object area. For these reasons, the training preparation of our own UAV dataset is the first step and then the work of generalizing SegNet-based DL algorithm to the actual distribution of dataset in riverside of Chongqing is followed.

**Figure 3:**
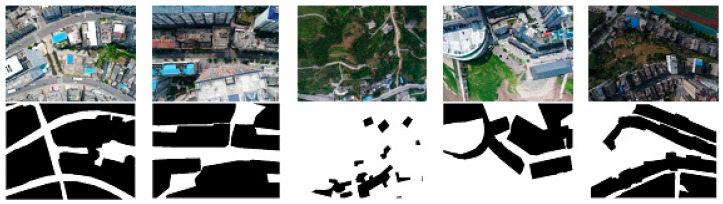
UAV images and their corresponding annotation images.

As to the UAV dataset production, we first separated all images used for training into two semantic classes and labeled them as building and non-building. The challenges in creating such datasets lie in that there are so many building patterns, different flight altitudes, confusing details caused by low attitude, and complex land cover types in the object area. The sample UAV dataset is shown in Figure 3. There are 2220 images included in this dataset. Each image consists of an original RGB image collected from UAV and labeled image with two semantic classes identified, and they are linked by the same ID. In our experiment, we divide these annotated datasets into three sets: 2000 sets for training, 20% of randomly selected from the training set for validating, and 220 sets for testing. The testing set is captured from different areas compared to the training sample set.

### 2.2. SegNet-Based Semantic Segmentation

One of the first end-to-end DL semantic segmentation approaches is the FCN model, which is a convolutional neural network and a reverse step with an encoder-decoder architecture. The encoder is topologically identical to VGG-16 architecture in which convolutional and pooling layers are included. On the other hand, decoder has upsampling and deconvolution layers that are key to the DL semantic segmentation. The last decoder will produce the segmented output at the same resolution to the input image. Later, Noh et al. [20] improved the architecture by containing stacked deconvolution and unsampling layers, which outperforms FCNs but has a more complex training procedure. Furthermore, Badrinarayanan et al. [21] proposed SegNet architecture which reduced the learning process by reusing pooling indices in encoder layers to perform upsampling. The successful semantic segmentation using Caffe was introduced on the website [22]. SegNet has showed superior performance compared to other deep architectures such as DeepLab-LargeFOV, DeepLab-LargeFOV-denseCRF, FCN, FCN (learn deconv), and DeconvNet by CamVid and SUNRGB-D dataset [21,23,24]. Moreover, the SegNet network architecture has been used as the standard architecture for new architecture optimization [12,25,26,27]. Based on all these achievements, the SegNet network architecture was selected in this research.

**Figure 4:**
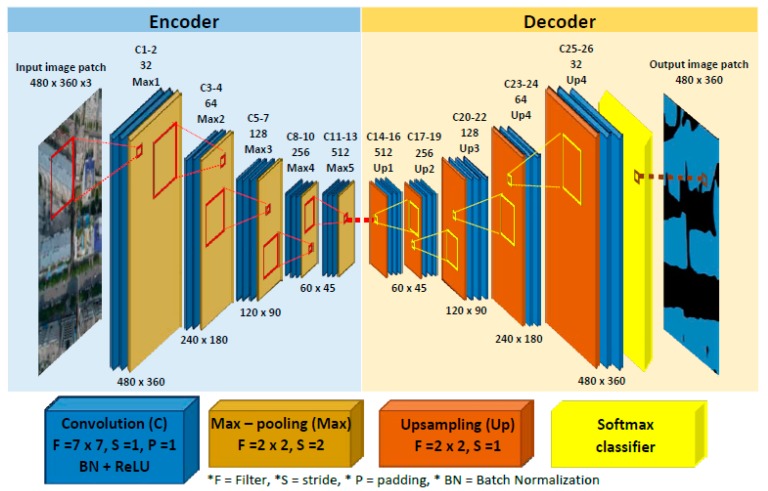
SegNet architecture for semantic labeling.

The SegNet architecture is illustrated in Figure 4, which is comprised by the encoder network, decoder network, and the final being pixel-based classification layer. The encoder network consists of 13 convolution layers and max-pooling layers which translate invariance over small spatial shifted images. Each convolution will be convoluted with filters to produce feature maps and batch normalization, then it will be applied to Rectified Linear Unit (ReLU) to activate the function for conducting the non-linear transformation of the feature map to the system. While the decoder network consists of 13 convolution layers and upsampling layers, and the first layer is to decode sparse feature map by max-pooling indices as called upsampling layer, then the output layer will be convoluted with decoder filter bank to produce dense feature maps. The final decoder is fed to classification method. The classifiers in DL can be divided into linear and nonlinear, and mainly linear classifiers such as Support Vector Machine and SoftMax are used in DL [28]. In this research, the soft-max classifier is selected to predict maximum probability at pixel level according to the number of classes.

The learning process is defined by the tuning weights in the convolutional layers using the back-propagation algorithm. The numbers of layers and parameters in each layer are set to the same as SegNet architecture in [21]. To prevent overfitting, this experiment requires a large amount of training data and several function settings such as data augmentation (more tolerant to the position and orientation), early stopping technique (to interrupt training and to evaluate the model on a validation set at regular intervals i.e., every 50 steps). In the study, L2 regularized logistic regression which used LIBLINEAR to cost function was added, and the dropout was set to 0.5 which was added to the end of deeper convolutional layer, together with batch normalization layers [29] being added to every convolutional layer.

Fine-tuning of the hyperparameters are necessary for the supervised learning process. The implementation of training network is based on the standard architecture with stochastic gradient descent (SGD) at a learning rate of 0.001 and reduced by a factor of 10 every 50 iterations. The total number of iterations is 50,000. The remaining two hyper-parameters, momentum and weight decay are set to 0.9 and 0.0005, respectively. The model training process is implemented on a standard desktop with CPU Intel Core i7 (3.4GHz), RAM 48GB, GPU NVIDIA Quadro K620, and 2 GB memory. All experiments are developed based on TensorFlow library with Python 2.7.

### 2.3. Methods

To examine the efficiency of above architecture when it was applied to a pure UAV dataset in detecting and extracting buildings, two steps were followed in this paper: Step 1—examining the building extraction accuracy from UAV images and Step 2—evaluating the performance of supervised learning procedure on two open standard datasets having different building patterns and styles as well as geographic locations. The main aim of designing Step 2 is to verify the validity and advantage of the SegNet architecture used. Details are described in the following sections. In all experiments, the training process is evaluated by building extraction accuracy assessment and the results are detailed in the experimental results section.

#### 2.3.1. Step 1: To Examine the Building Extraction Accuracy from UAV Images

In order to detect and extract buildings along the river bank area in Chongqing City, China, the transferability of learned features was examined by training with image samples from 16 flights covering the entire object area and was tested on another two flights of other locations (area_1 and area_2), while area_1 represents the crowded area with high buildings and area_2 represents a fairly sparse area along riverside with low buildings. The count of image patches corresponding to the UAV dataset is provided in Table 1.

The original size of the UAV images used in our work (5472 × 3648 pixels) cannot be input to the network architecture due to the insufficient hardware memory (GPU). Therefore, a resizing operation is necessary. The expected image size is 480 × 360 pixels. The reason for choosing this image size is that the original SegNet architecture designed by Badrinarayanan et al. [21] uses an image with a size of 480 × 360 pixels as the input. To evaluate the feasibility of this network architecture on UAV dataset, it is better to keep the same image size to avoid any systematic bias.

#### 2.3.2. Step 2: To Evaluate Supervised Learning Procedure on Two Open Standard Datasets

**Figure 5:**
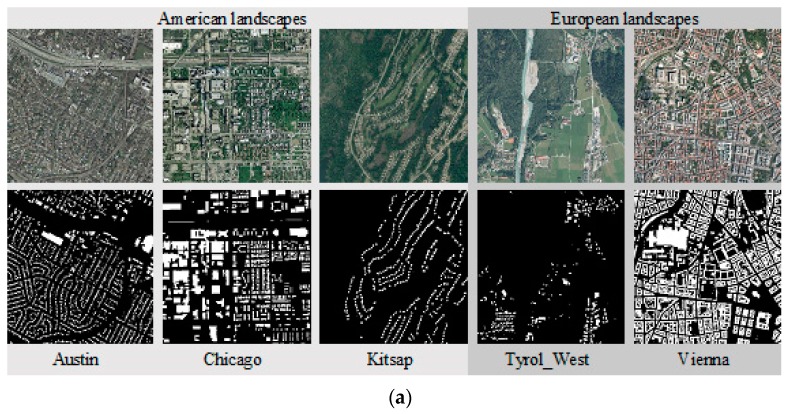

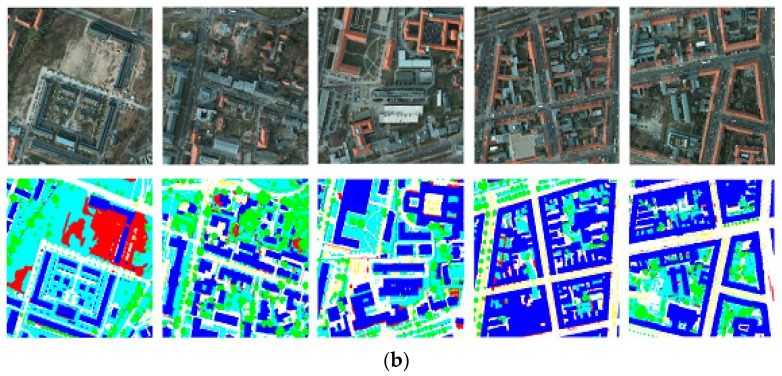
Two open standard datasets used: (**a**) Inria Aerial Image Labeling Dataset; (**b**) ISPRS Potsdam semantic labeling dataset.

To justify the feasibility of proposed DL semantic segmentation procedure on building extraction from high-resolution images, similar experiments were performed on another two standard datasets: Inria Aerial Image Labelling dataset (Inria aerial dataset) [30] and ISPRS Potsdam semantic labeling dataset (ISPRS Potsdam dataset) [31]. The Inria aerial dataset is the open benchmark dataset released by USGS through the National Map Service (nationalmap.gov), which was collected over the United States (US) and Austria. It covers a variety of urban landscapes from Austin (TX), Chicago (IL), Kitsap County (WA)), Tyrol and Vienna as shown in Figure 5a. This open access dataset is available online [32]. ISPRS Potsdam dataset is very high-resolution aerial photograph provided by commission III of ISPRS, and images in this dataset were captured over the city of Potsdam in Germany, where there are very dense settlement structures as shown in Figure 5b. This dataset is also available online [33].

The Inria aerial dataset has 24 densely annotated image tiles with a high spatial resolution of 10–30 cm and an image size of 6000 × 6000 pixels, while in our experiment 20 tiles were used for training and the other four tiles are for testing. As to ISPRS Potsdam dataset, it has 36 image tiles with a spatial resolution of 5 cm and an image size of 1500 × 1500 pixels and 30 image tiles were adopted to form the training set, and the remaining six tiles were used for testing. Validation sets were randomly selected from training sets with a ratio of 20%. The annotated image consists of two classes: building and non-building. These image tiles are clipped and split to 480 × 360 pixels. The numbers of image patches corresponding to each dataset are provided in Table 2.

### 2.4. Accuracy Assessment

Indexes are used to evaluate the performance of DL semantic segmentation. The first is Overall Accuracy which is achieved by calculating the summary of correctly classified pixels, the second is Mean Intersection over Union (mIoU) which computes the average of IoU. The IoU is computed from a ratio between the union and intersection of ground truth and predicted segmentation. The ratio is calculated from intersection value (the number of true positives) divided by union value (the sum of true of false positives, false negatives, and true positives).

## 3. Experiment Results

### 3.1. Experiment 1: Building Extraction Based on Trained UAV Dataset

This experiment is to evaluate the performance of supervised learning model for detecting and extracting buildings based on the UAV datasets. During the training process, the last validation step is set to 50,000 iterations. The accuracy results of the proposed SegNet architecture is summarized in Table 3. It is seen that a very good performance is achieved with an overall accuracy of 92.47% and mIoU of 84.39%, respectively. The numerical evaluations of this model are done based on two testing sets: area_1 and area_2. Apparently, SegNet also achieves a very good performance with the overall accuracy in area_1 (92.59%) and area_2 (89.50%). The distribution accuracy of mIoU in both object areas is much closer (0.3% difference). It is meaning that the performance of extracted building and non-building overlapped with the reference building is fair. However, if we compare the classification accuracy in each class, it is shown that the building class in area_2 has building class accuracy 90.59% which is higher accuracy than area_1 by 6.47%, while for non-building class, the accuracy of area_1 is 93.59% which is higher than area_2 around 5.24%.

**Figure 6:**
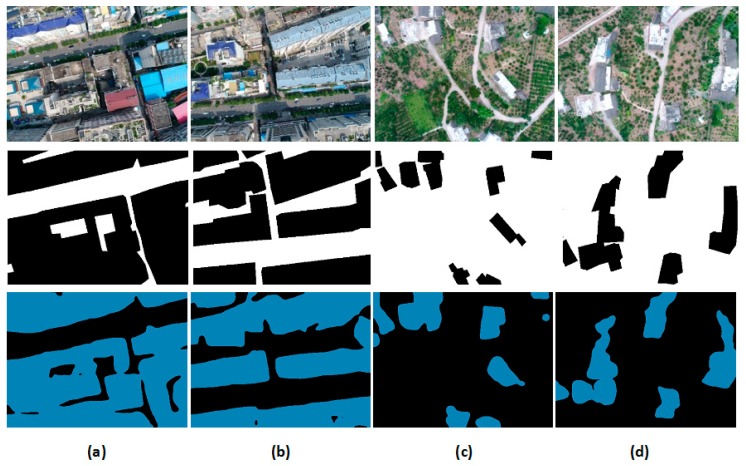
Visual segmentation results on two testing sets which consist of input RGB image (top), reference data (middle), and building extraction result (bottom). (**a**,**b**) are from testing area (area_1); (**c**,**d**) are from testing area (area_2).

Visual comparisons (Figure 6) show that the segmentation results of SegNet architecture are more accurate and coherent. For example, in Figure 6a,b, the buildings in the urban area (area_1) which have complex building shapes and patterns are detected correctly, even though there are gaps between buildings that are mislabeled, but because roof lawns and cement roofs are similar to garden and cement road, and the shape and size are not detected with the very high accuracy. In Figure 6a, it is seen that the SegNet architecture can segment the building gaps, but it cannot detect a good building shape even though it shows clear building features. In contrast, the representation of area_2 (suburban) in Figure 6c,d shows that the small buildings are completely detected.

### 3.2. Experiment 2: Building Extraction from Two Standard Datasets

**Figure 7:**
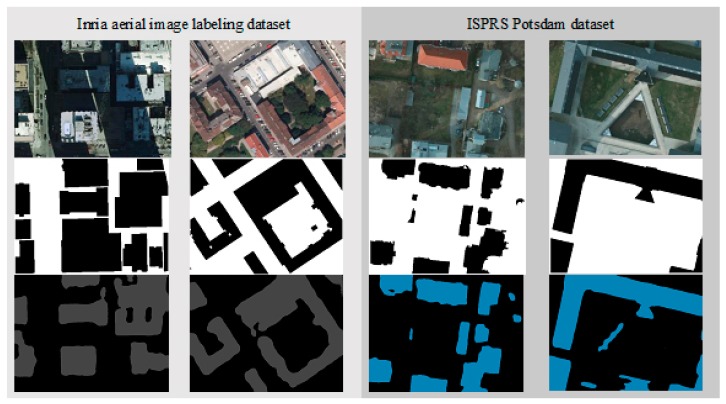
Visual segmentation results on Inria aerial dataset (**left**) and ISPRS Potsdam dataset (**right**) which consist of input color image (**top**), reference segmentation data (**middle**), and building extraction result (**bottom**).

Another experiment is also made by applying the proposed SegNet-based DL procedure on two open standard high-resolution images to extract buildings. The experiment results are shown in Table 4. The Inria aerial dataset achieves an overall accuracy of 93.42% and an 85.32% mIoU, while ISPRS Potsdam dataset achieves an overall accuracy of 95.79% and mIoU at 87.80%. In the building class, two standard datasets have high classification accuracy up to 91.40% of Inria dataset and 92.12% of ISPRS dataset. The visual building extraction results from both datasets are shown in Figure 7. The building extraction accuracies are 91.40% for Inria aerial image labeling dataset and 92.12% for ISPRS Potsdam dataset.

## 4. Discussion

The results mentioned in Section 3 show that SegNet-based deep learning approach works well on the UAV dataset in extracting buildings along the riverbank with fairly good accuracy. The buildings in UAV images are detected accurately, even though there exist complex features, multiple building patterns, and various styles. In two testing areas, both high and small buildings are detected correctly, and the average overall accuracy of both test areas (area_1 and area_2) for building extraction is nearly 90%. The area_2 has better performance than area_1, and that is because buildings in this area are mainly small building with a simple structure and few styles. Another reason is that there are larger percentages of bare land and vegetation in area_2, which have different features and patterns from buildings. However, there still has some error in classifying buildings and the ground due to the smooth features of these objects and less difference in the height dimension. As to area_1, there are much richer features inside, which is almost filled with the built-up area, and the medium and high buildings in area_1 have more structural complexities which will cause more classification errors. However, when we compare the building classification accuracy, it shows that the supervised training can classify high and medium building more accurately, while the classification accuracy drops when it is used to classify small building in area_2. This is because that there are a variety of high building features and a huge of data sample in the city, on the other hands, there is a smaller number of small building data sample included in our UAV dataset. Enlarging training samples may be helpful to improve segmentation result. Though experiments showed that deep learning in our work can detect all building visually, it is not always correct in extracting building shapes, and building shade or ground are easily mislabeled as buildings. Furthermore, modern architectures, roof lawns, cement roofs, and also shadows are difficult to correctly classify from the two-dimensional images. Adding some other features like three-dimensional features may work better [34]. We will continue our study in this field.

The classification accuracy of Inria aerial dataset is almost the same as reported in [30]. The experiment results on the ISPRS dataset also show the performance as good as claimed in [16,25], and a better overall accuracy and building classification than the results described in [18]. All these experiments confirm that SegNet-based DL semantic segmentation can be used as a baseline for building extraction from high-resolution remote sensing images.

## 5. Conclusions

This paper evaluated the performance of SegNet-based deep learning on detecting and extracting buildings from UAV images, and its feasibility and advantage have been confirmed. The supervised training model based on SegNet architectures shows very good performance with an average overall accuracy of nearly 90% in extracting buildings from the testing datasets which are collected from other locations than the training location. The experimental results on two standard datasets (ISPRS and Inria aerial datasets) also confirm that the proposed DL procedure is suitable for building extraction from very high-resolution images. Further research will be conducted on improving the network architecture to get better results.

## Figures and Tables

**Table 1:** The number of samples for training, validating, and testing from our UAV datasets.

Dataset	Training	Validating	Testing	
			Area_1	Area_2
UAV dataset	1600	400	120	100

**Table 2:** The number of samples for training, validating, and testing from the two standard datasets.

Dataset	Training	Validating	Testing
Inria Aerial Image Labeling Dataset	23,100	3850	770
ISPRS Potsdam semantic labeling dataset	320	80	80

**Table 3:** Classification accuracy results of UAV dataset (%).

Dataset	Building	Non-Building	mIoU	Overall Acc.
Numerical evaluation on validating set				
UAV dataset	92.01	94.67	84.39	92.47
Numerical evaluation on two testing sets				
Area_1	84.12	93.59	81.27	92.59
Area_2	90.59	88.35	80.97	89.50

**Table 4:** Classification accuracy results of two standard datasets (%).

Dataset	Building	Non-Building	mIoU	Overall Acc.
Inria aerial image labeling dataset	91.40	94.84	85.32	93.42
ISPRS Potsdam dataset	92.12	96.65	87.80	95.79
